# Improving the Thickness Uniformity of Micro Gear by Multi-Step, Self-Aligned Lithography and Electroforming

**DOI:** 10.3390/mi14040775

**Published:** 2023-03-30

**Authors:** Huan Wang, Jing Xie, Tao Fan, Dapeng Sun, Chaobo Li

**Affiliations:** 1 Institute of Microelectronics of the Chinese Academy of Sciences, Beijing 100029, China; 2 University of Chinese Academy of Sciences, Beijing 100049, China

**Keywords:** micro-electroforming, thickness uniformity, self-aligned lithography, roughness

## Abstract

The thickness nonuniformity of an electroformed layer is a bottleneck problem for electroformed micro metal devices. In this paper, a new fabrication method is proposed to improve the thickness uniformity of micro gear, which is the key element of various microdevices. The effect of the thickness of the photoresist on the uniformity was studied by simulation analysis, which showed that as the thickness of the photoresist increased, the thickness nonuniformity of the electroformed gear should decrease due to the reduced edge effect of the current density. Differently from the traditional method performed by one-step front lithography and electroforming, multi-step, self-aligned lithography and electroforming are used to fabricate micro gear structures in proposed method, which intermittently keeps the thickness of photoresist from decreasing during processes of alternate lithography and electroforming. The experimental results show that the thickness uniformity of micro gear fabricated by the proposed method was improved by 45.7% compared with that fabricated by the traditional method. Meanwhile, the roughness of the middle region of the gear structure was reduced by 17.4%.

## 1. Introduction

In recent years, micro-electroforming has become important technology for the fabrication of micro-metal devices in the field of microelectronics and microelectromechanical systems (MEMSs) [1]. A combination of lithography technology and electrodeposition makes it possible to fabricate metal microstructures suitable for micro-molds, micro-sensors and micro-actuators [2,3,4], which is a method that has the advantages of high precision, a wide range of processing sizes and mass production [5]. However, the thickness nonuniformity of the electroformed layer is a bottleneck problem in the fabrication of microdevices using micro-electroforming technology [5,6,7], which affects the usability of micro metal devices. Until now, post-processing such as lapping/polishing has been used to ensure dimensional accuracy and surface quality, but this exacerbates the fabrication efficiency and cost. Therefore, it is of great significance to study the methods for improving the thickness uniformity.

So far, several methods have been used to improve the thickness uniformity, mostly including optimizing electroforming parameters [8], adding additives [9], using pulse or reverse-pulse current [10], using a copying anode [11], using an assistance electrode and shield [1,12,13], ultrasonic electrodepositing [14,15] and megasonic agitation [5]. The shape of the cathode depends on the photoresist mold, which is critical to the quality of the electroformed structure. The shape of the photoresist mold has been studied by using a coplanar auxiliary cathode to improve the thickness uniformity of electroformed mold [16]. The thickness of photoresist has been studied through simulation analysis and experimental verification that the high aspect ratio photoresist restricts the mass transfer during the electroforming process [17]. However, the thickness of the photoresist is rarely used to study its effect on the uniformity.

Micro gears are the key elements of various micro-systems and devices used for operating their actuators, reducers and driving components in the fields of micro-motors, micro-pumps, micro-motors, robotics, etc. [18]. Many scholars have studied the fabrication of micro-gear structures using the micro electroforming technology [19,20,21,22]. One of main engineering limitations is nonuniform thickness [19]. Therefore, aiming at the effect of photoresist thickness on the thickness uniformity, a multi-step, self-aligned lithography and electroforming method is proposed to improve thickness uniformity of the micro gear in the process of micro electroforming.

## 2. Experimental

### 2.1. Simulation

#### 2.1.1. Geometric Model

**Figure 1:**
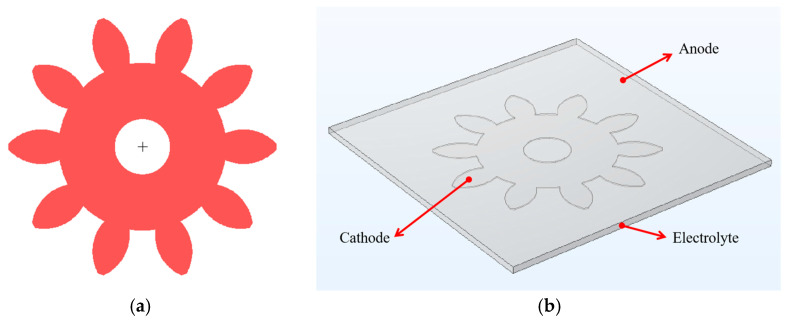
(**a**) Plane structure diagram of the micro gear; (**b**) geometric model.

Figure 1a presents the plane structure diagram of a micro gear which has the gear diameter of 960 µm, 10 teeth and a modulus of 0.08 mm. The simulation was performed using COMSOL Multiphysics. The geometric model is shown in Figure 1b, which includes the anode plane, the electrolyte domain and the cathode. The anode is the Ni plate, so it is simplified as a plane. The cathode is simplified as the trench of the micro gear structure, which also is the electroforming surface. The sidewall of micro gear structure is a vertical photoresist wall. In order to compare the effect of photoresist thickness on uniformity, the thickness of photoresist layer (TP) was set as 5, 10, 15, 20, 25 or 30 µm, and the time of electroforming was set as 180 s. 

#### 2.1.2. Electroforming Model

The electric field in the electrolyte can be described as [1,16]

(1) $$i_{l}=-\sigma \nabla \phi_{l}$$

(2) $$\nabla \cdot i_{l}=0$$

where $i_{l}$ is current density (A/m^2^) in the absence of the concentration gradients in the electrolyte, σ is the conductivity of the electrolyte (S/m) and $\phi_{l}$ is the electrolyte potential (V).

The Bulter–Volmer expression is used to describe the electrode reaction kinetics for the cathode surfaces [1,16].

(3) $$i_{\mathrm{loc}}=i_{0}\left(\exp\left(\frac{a_{a}F\eta }{\mathrm{RT}}\right)-\exp\left(\frac{-a_{c}F\eta }{\mathrm{RT}}\right)\right)\;$$

where $i_{\mathrm{loc}}$ is current density (A/m^2^) on the cathode’s surface due to electrode reaction $;i_{0}$, $a_{a}$, $a_{c}$, F, R and T are exchange density (A/m^2^), anode transfer coefficient, cathode transfer coefficient, Faraday constant (C/mol), universal gas constant (J/(mol·K)) and temperature (K), respectively. η is overpotential (V) and is defined by [1,16]:

(4) $$\eta =\phi_{s}-\phi_{l}-E_{\mathrm{eq}}$$

where $\phi_{s}$ and $E_{\mathrm{eq}}$ are the potential of cathode surfaces (V) and equilibrium potential (V), respectively. The initial values of $\phi_{s}$ and η are both 0 V. Thus, from the equation, the initial condition for $\phi_{l}$ is as follows:

(5) $$\phi_{l}=-E_{\mathrm{eq}}$$

The boundary condition of total current is used for the electroforming area:

(6) $$I_{t}=-i_{\mathrm{avg}}S$$

where $I_{t}$ is total current (A), ‘−’ means that electrons outflow from the electrode, $i_{\mathrm{avg}}$ is the average current density of the cathode (A/m^2^) and S is the total surface area of the electroforming layer (m^2^).

Based on Faraday’s law, the nickel depositing velocity can be described as [1,16]:

(7) $$V_{\mathrm{dep}}=\frac{\mathrm{MN}}{\rho }=-\frac{i_{\mathrm{loc}}}{F\;}\frac{\gamma M}{n\rho }$$

where $V_{\mathrm{dep}}$ is the depositing velocity (m/s), M is the molar mass of nickel (kg/mol), γ is the stoichiometric coefficient, n is the electron number of the reaction and ρ is the density of nickel (kg/m^3^).The simulation parameters are shown in Table 1.

### 2.2. Experimental Conditions

Electroforming was carried out by electroforming equipment (Yamamoto-MS, A-52-ST6A-100B, Tokyo, Japan). The formula of electroforming solution was: Ni[NH_2_SO_3_]_2_·4H_2_O (400 g·L^−1^), NiCl_2_ (20 g·L^−1^), H_3_BO_3_ (10 g·L^−1^) and wetting agent (5 g·L^−1^). The operating temperature was 45 °C, the pH value was about 4.0 and the current density was 1 A/dm^2^. 

Indium tin oxide (ITO) glass was used as the base material, which has the advantages of mature fabrication technology and low cost. At the same time, ITO glass has the functional advantages of certain light transmittance and conductivity, so it could realize back self-aligned lithography, which could avoid alignment problems that may be introduced by multi-step lithography.

### 2.3. Experimental Methods and Processes

**Figure 2:**
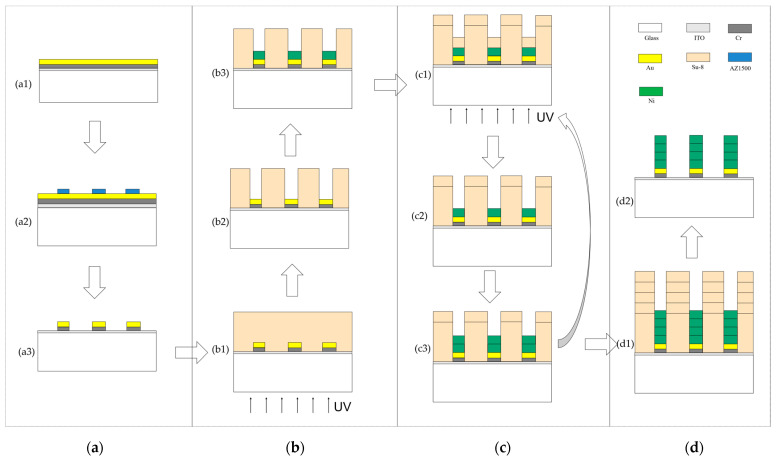
Fabrication processes of microstructure in the proposed fabrication method. (**a**) Prepare the patterned seed layer: (**a1**) Cr/Au seed layer, (**a2**) front lithography and development of AZ1500 photoresist, (**a3**) removal of AZ1500 photoresist; (**b**) first back-alignment lithography and electroforming: (**b1**) spin SU-8 and back-alignment lithography, (**b2**) development, (**b3**) electroforming; (**c**) repeated back-alignment lithography and electroforming to achieve the required thickness of the electroforming layer: (**c1**), (**c2**) and (**c3**), respectively, are repetitions of (**b1**), (**b2**) and (**b3**); (**d**) removal of photoresist: (**d1**) the result of multi-step back-alignment lithography and electroforming, (**d2**) removal of the SU-8 photoresist.

**Figure 3:**
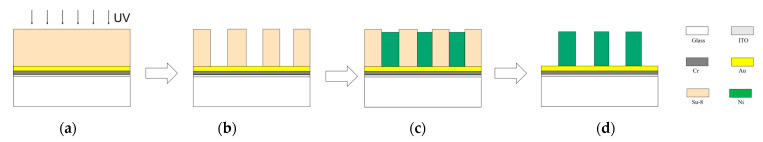
Fabrication processes of microstructure in the traditional fabrication method. (**a**) Cr/Au seed layer, spin SU-8 and front lithography; (**b**) development; (**c**) electroforming; (**d**) removal of the SU-8 photoresist.

A multi-step, self-aligned lithography and electroforming method was proposed to improve the thickness uniformity of a micro electroforming layer, as shown in Figure 2. For comparison, the traditional micro electroforming method is shown in Figure 3, which was performed by one-step front lithography and electroforming [23,24].

#### 2.3.1. Fabrication Process of the Traditional Fabrication Method

The fabrication process’s diagram is shown in Figure 3. An SU-8 2030 photoresist was spun on a Cr/Au (10 nm/100 nm) seed layer for a pre-spin of 500 rpm/10 s and a main spin of 3000 rpm/50 s. The thickness was about 30 µm. A soft bake (65 °C/60 s + 95 °C/5 min) on a contact hotplate was implemented. After soft baking, the resist was cooled down for 10 min to room temperature. Then, the front was exposed to the UV light (365 nm) at 6 mW/cm^2^ for 20 s. A post-exposure bake (65 °C/60 s + 95 °C/5 min) was carried out on a contact hotplate, and the resist was cooled down to room temperature. Then, the photoresist was developed in SU-8 developer. After development, O^2^ plasma could be used (100 W, 50 sccm, 60 s) to remove any remaining residue, and an SU-8 photoresist mold was obtained. Finally, after electroforming 80 min, the SU-8 photoresist was immersed in the SU-8 remover to obtain the microstructure. Optionally, O^2^ plasma could be used (100 W, 50 sccm, 2 min) to remove any remaining residue.

#### 2.3.2. Fabrication Process of the Proposed Fabrication Method

The fabrication process’s diagram is shown in Figure 2. In order to improve the adhesion between the subsequent micro electroforming layer and the substrate, and form a self-aligning metal shielding layer, the first step in the fabrication of the microstructure is to prepare the patterned seed layer. Under ultrasonic vibration, the substrate was washed successively with acetone, ethanol and deionized water for 10 min; then dried with nitrogen; and finally, dehydrated and baked in a 110 °C convection oven for up to one hour. After substrate cleaning, Cr/Au (10 nm/100 nm) was deposited by magnetron sputter on the substrate, and then a layer of a common positive photoresist (AZ1500) was spun on the substrate for a pre-spin of 500 rpm/10 s and a main spin of 3000 rpm/50 s. The thickness of the photoresist layer was about 1.5 µm. The photoresist was prebaked at 110 °C for 1 min, and then was exposed to the UV light at 6 mW/cm^2^ for 30 s after cooling for 5 min to room temperature. Then, the photoresist was developed in AZ1500 developer. Finally, the seed layer was obtained after removing 100 nm Au film, 10 nm Cr film and photoresist by wet etching. 

The first back-alignment lithography and electroforming were performed using the patterned metal shield formed by the seed layer. The photoresist and its lithography parameters used were the same as those mentioned in the traditional method, except that the exposure method was performed on the backside of the swatch. The thickness of the photoresist was also about 30 µm. After back lithography and development, O^2^ plasma could be used (100 W, 50 sccm, 60 s) to remove any remaining residue. Finally, electroforming of 20 min was performed, and the thickness of electroforming layer was about 4 µm.

In order to keep the thickness of the photoresist at 30 µm after the electroforming in the previous step, the new photoresist was used to perform the back-alignment photolithography again. The increased thickness of the photoresist should be consistent with the increased thickness of the electroformed layer. The SU-8 2005 photoresist was spun in a pre-spin of 500 rpm/10 s and a main spin of 3300 rpm/50 s. The thickness was about 4 µm. A soft bake (95 °C/2 min) on a contact hotplate was implemented. After soft baking, the resist was cooled down for 5 min to room temperature. Then, the back was exposed to the UV light at 6 mW/cm^2^ for 26 s. A post-exposure bake (95 °C/2 min) was carried out on a contact hotplate, and the resist was cooled down to room temperature. Then, the photoresist was developed in SU-8 developer. After development, O^2^ plasma could be used (100 W, 50 sccm, 60 s) to remove any remaining residue, and the new photoresist mold of about 30 µm was obtained. The electroforming of 20 min was performed, and the thickness of the electroforming layer was about 4 µm. In order to make the total electroforming time consistent with that of the traditional method, the above lithography and electroforming process was repeated three times. The back was exposed to the UV light at 6 mW/cm^2^ for 26 s, 27 s, and 28 s, respectively.

The SU-8 photoresist was stripped in SU-8 remover at 80 °C for 60 min to obtain the microstructure. Optionally, O^2^ plasma could be used (100 W, 50 sccm, 2 min) to remove any remaining residue.

### 2.4. Measurements

Nonuniformity α was used to quantify thickness uniformity of the electroformed layer. It is defined by [2,16]

(8) $$\alpha =\frac{h_{\max}-h_{\min}}{h_{\min}}\times 100\%$$

where $h_{\max}$ and $h_{\min}$ are the maximum and minimum thickness of the electroformed layer, respectively.

The thickness distribution and current-density distribution were obtained by COMSOL simulation [1,16]. The morphology was measured by field emission scanning electron microscopy (FE-SEM, Hitachi, S4800, Ibaraki, Japan). The microstructure profile, thickness and roughness were measured by a laser scanning confocal microscope (LSCM, Olympus, Tokyo, Japan, OLS4000). 

## 3. Results and Discussion 

### 3.1. Simulation

**Figure 4:**
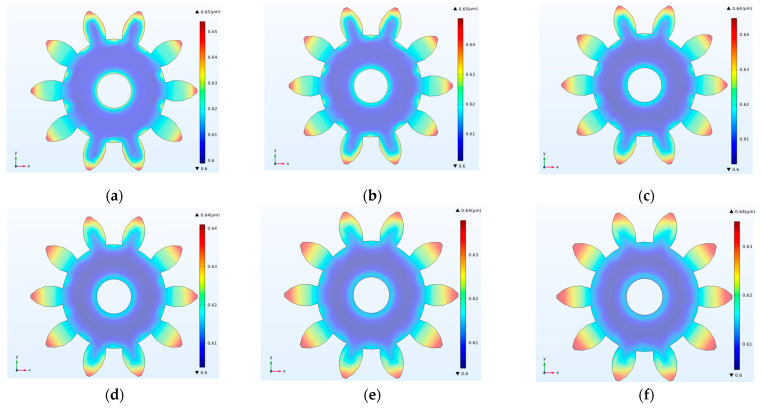

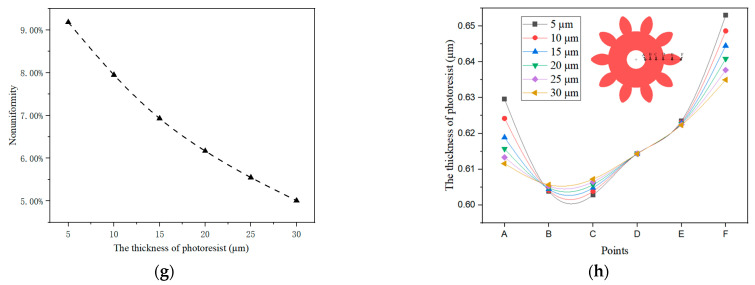
Simulation results and calculated results of thickness distributions with different thicknesses of the photoresist. (**a**) TP = 5 µm; (**b**) TP = 10 µm; (**c**) TP = 15 µm; (**d**) TP = 20 µm; (**e**) TP = 25 µm; (**f**) TP = 30 µm; (**g**) Calculated nonuniformity for different TPs; (**h**) thickness distribution curves of location points (A, B, C, D, E and F) for different TPs.

Figure 4a–f show the six groups of simulation results about thickness distributions under different TPs. The results showed that the thickness distributions under different conditions were consistent, and the thickness of the gear edges was much larger than that of the middle region. The nonuniformity was calculated, and the results are shown in Table 2 and Figure 4g. The nonuniformity was a minimum of 5.01%, which meant that electroformed gear had the best thickness uniformity with changing TP. The corresponding best value was 30 µm. It was revealed that as the thickness of photoresist became larger, the nonuniformity of the electroformed layer became smaller. A, B, C, D, E and F are six points from the inner edge of the gear to the outer edge. The thickness values were extracted from the six locations in Figure 4a–f, respectively, and the thickness-distribution curves for different TPs were obtained, as shown in Figure 4h. This shows that the smaller the thickness of photoresist, the farther the thickness values at the edge points (A and F) of the gear structure deviated from the middle points (B, C, D and E), which might lead to greater nonuniformity, as shown in Figure 4g.

**Figure 5:**
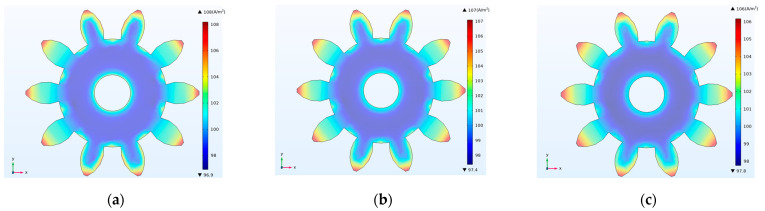

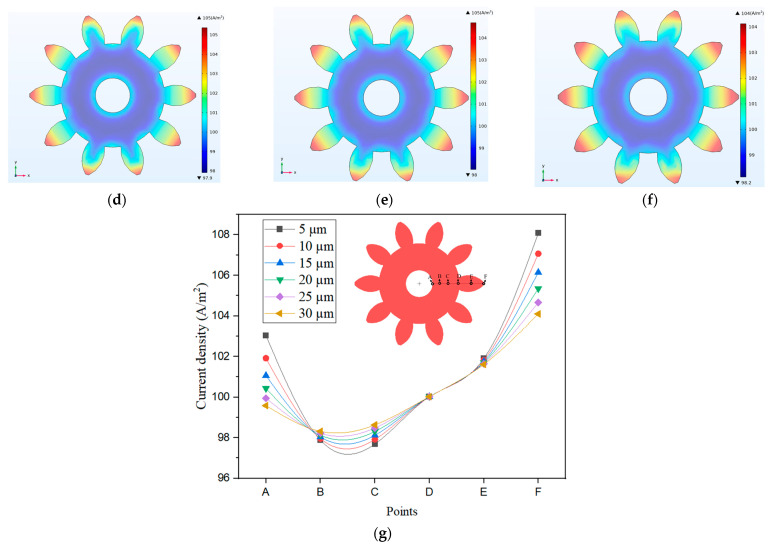
Simulation results of current-density distributions for different thicknesses of photoresist. (**a**) TP = 5 µm; (**b**) TP = 10 µm; (**c)** TP = 15 µm; (**d**) TP = 20 µm; (**e**) TP = 25 µm; (**f**) TP = 30 µm; (**g**) current-density distribution curves of location points (A, B, C, D, E and F) for different TPs.

According to Faraday’s law, the thickness of the electroforming layer is proportional to the current density, so the current-density distribution on the surface of the gear structure can be used to analyze its thickness distribution [16]. Figure 5a–f show the current-density distributions under different TPs. The results show that the current density at the peripheral edges of the gear structure was larger than that of the middle region, indicating that the edge effect of the current was obvious. The edge effect of the outer edge was larger than that of the inner edge. Figure 5g shows the current density curves of the same six points (A, B, C, D, E and F) as those in Figure 4g under different TPs. It was found that as the photoresist thickness increased, the current density at the edge points (A and F) was closer to that at the middle points (B, C, D and E), and the edge effect decreased, which might be the reason why the thickness nonuniformity became smaller and smaller. 

### 3.2. Experiment

**Figure 6:**
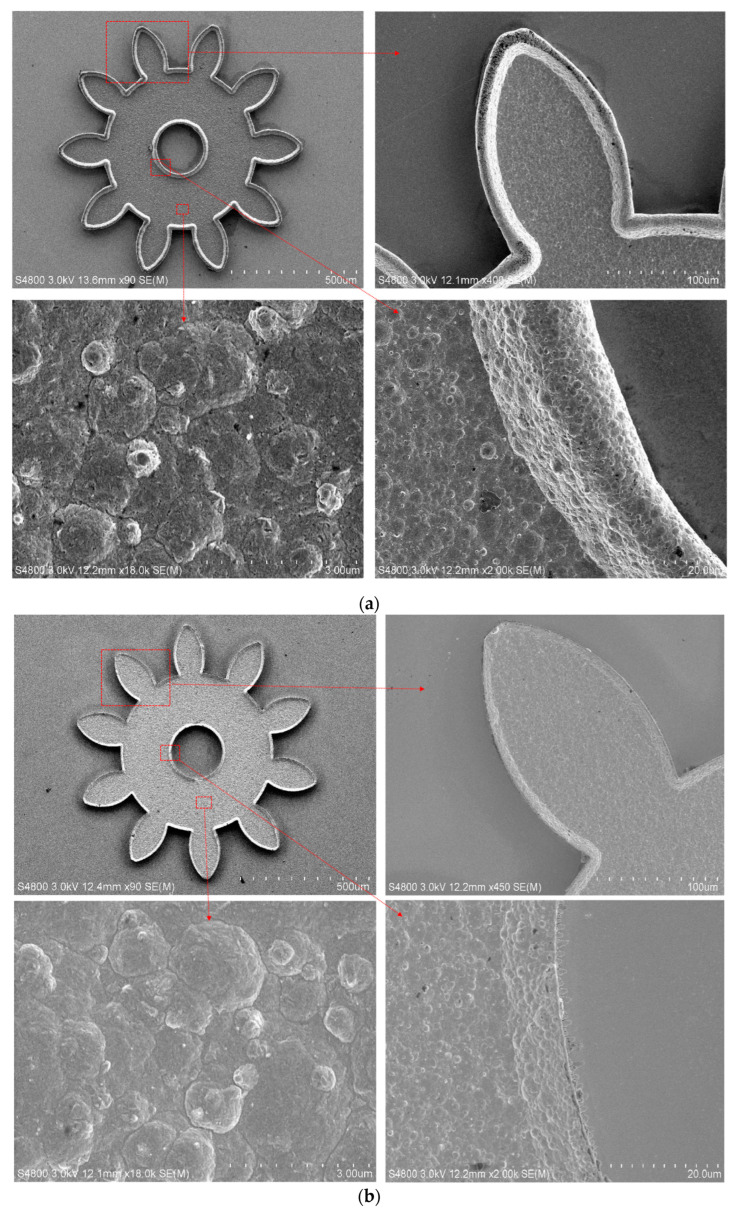
FE-SEM images of fabricated micro gear structures. (**a**) FE-SEM images in the traditional method; (**b**) FE-SEM images in the proposed method.

Figure 6 shows the FE-SEM photos of micro gear structures fabricated by the traditional method and the proposed method. The bulges on the inner edge and outer edge of the gear electroformed by the proposed method were more prominent than those of the gear made by the traditional method. From the FE-SEM microphotos of the middle region, it was observed that there was no apparent difference in the two methods’ results.

**Figure 7:**
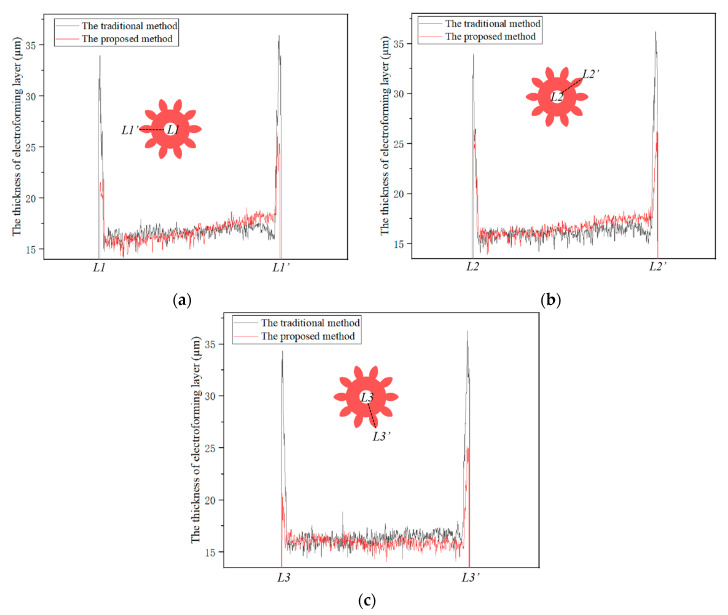
The thickness distributions of three dotted lines (L1-L1′, L2-L2′ and L3-L3′) after the use of two methods. (**a**) The thickness curves at the locations of L1-L1′; (**b**) the thickness curves at the locations of L2-L2′; (**c**) the thickness curves at the locations of L3-L3′.

The thickness distributions of the three dotted lines (L1-L1′, L2-L2′ and L3-L3′) from the inner edge to outer edge were measured by LSCM, as shown in Figure 7. In two methods, all thickness values of the middle region were approximately 16 µm. The maximum thickness values of the inner edge were reduced from 34.00, 34.04 and 34.34 µm to 22.60, 26.22 and 20.69 µm by the proposed method, respectively. The maximum thickness values of outer edge were reduced from 35.93, 36.24 and 36.30 µm to 26.46, 27.90 and 25.13 µm by the proposed method, respectively. It could be seen that the thickness of the gear edges generated by the proposed method was much lower than that of the traditional method, indicating that the proposed method could improve the edge effect of the electroformed gear. At the same time, the thickness of the outer edge of the gear was higher than that of the inner edge, which was consistent with the simulation of the current density distribution under different TPs. The thickness nonuniformity of three dotted lines in the two methods is shown in Table 3. It was found that the nonuniformity values were reduced from 153.83%, 157.48% and 153.02% to 85.00%, 88.30% and 78.60% by the proposed method, respectively. Thus, the average nonuniformity was reduced by 45.7%, which indicates that the proposed method could improve the uniformity of fabricated micro gears. 

**Figure 8:**
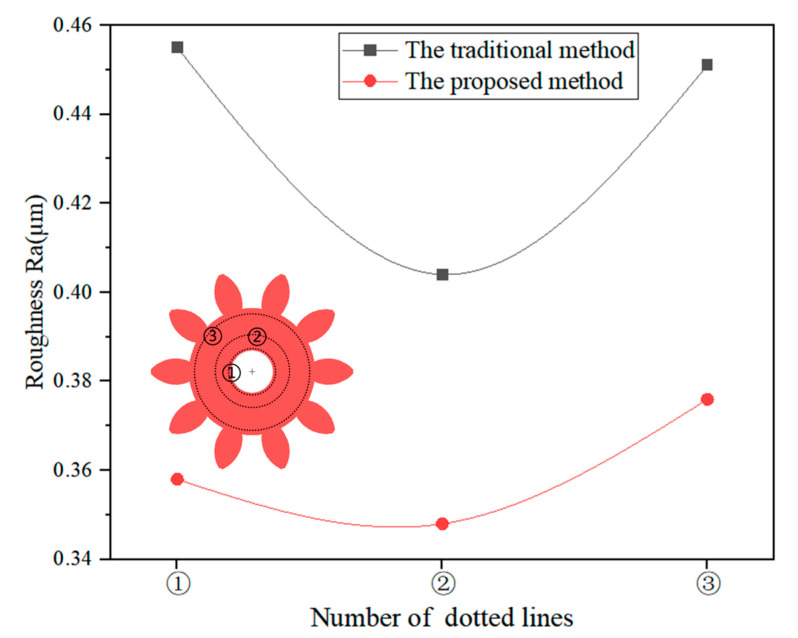
The roughness levels at different dotted lines on the gears fabricated by two methods.

Meanwhile, the roughness (Ra) values of the three dotted lines (①, ② and ③) in the ring area of each gear were measured by LSCM, as shown in Figure 8. The roughness values were reduced from 0.455, 0.404 and 0.451 µm to 0.358, 0.348 and 0.376 µm by the proposed method, respectively. The average roughness was reduced by 17.4%, which indicates that the proposed method could reduce the roughness and was beneficial to improving the quality of the micro gear. 

In the proposed method, multi-step lithography makes the thickness of the photoresist mold remain at about 30 µm before each electroforming, which takes 20 min. In the traditional method, the thickness of the photoresist mold is continuously reduced as the thickness of electroforming is increased. The thickness of the photoresist before each 20 min electroforming procedure was equivalent to about 30, 26, 22 and 18 µm, respectively. Based on the results of the above simulations, it may be inferred that the nonuniformity of current density in the traditional method increases continuously and superimposes as electroforming continues; however, the proposed method intermittently keeps the photoresist thickness from decreasing through multi-step lithography and electroforming during the whole micro electroforming process, so the current density distribution of the gear is more uniform, and the edge effect is smaller. Thus, the proposed method can improve the uniformity of the electroformed micro gear and reduce its roughness.

## 4. Conclusions

In this paper, a multi-step, self-aligned lithography and electroforming method was proposed to improve thickness uniformity of the micro gear. The effect of the thickness of the photoresist mold on the thickness uniformity of the electroforming gear was studied by simulation analysis. Simulation results showed that increasing the thickness of photoresist can reduce edge effects and improve the uniformity of the micro gear. Compared with the gear structure fabricated by the traditional method, the edge effect of that fabricated by the proposed method was smaller, and the average nonuniformity was reduced by 45.7%. Meanwhile, the average Ra of the middle region of the gear structure was reduced by 17.4%. This method provides a new option for improving the thickness uniformity of a micro-electroforming metal gear.

## Figures and Tables

**Table 1:** Simulation parameters.

σ (S/m)	$i_{avg}$ (A/m^2^)	$a_{c}$	$a_{c}$	T (K)	$E_{eq}\;$ (V)	M (kg/mol)	ρ (kg/m^3^)	γ	n
0.95	100	1.5	0.5	318.15	−0.257	0.0586	8900	1	2

**Table 2:** Difference of α by TP.

TP (µm)	5	10	15	20	25	30
$h_{\max}$ (µm)	0.6539	0.6488	0.6447	0.6410	0.6378	0.6351
$h_{\min}$ (µm)	0.5989	0.6010	0.6029	0.6038	0.6043	0.6048
α	9.18%	7.95%	6.93%	6.17%	5.55%	5.01%

**Table 3:** Difference in α between two methods.

Line	L1-L1′		L2-L2′		L3-L3′	
Method	Traditional	Proposed	Traditional	Proposed	Traditional	Proposed
$h_{\max}$ (µm)	35.94	26.46	36.24	26.23	36.30	25.13
$h_{\min}$ (µm)	14.16	14.30	14.08	13.93	14.35	14.07
α	153.83%	85.00%	157.48%	88.30%	153.02%	78.60%

## Data Availability

Not applicable.
